# Self-assembled monolayers and titanium dioxide: From surface patterning to potential applications

**DOI:** 10.3762/bjnano.2.94

**Published:** 2011-12-20

**Authors:** Yaron Paz

**Affiliations:** 1 The Department of Chemical Engineering, The Russell-Berrie Institute of Nanotechnology and The Grand Water Research Institute, Technion, Haifa 32000, Israel

**Keywords:** photocatalysis, remote degradation, self-assembled monolayers, titanium dioxide

## Abstract

The ability to control the properties of self-assembled monolayers (SAMs) attached to solid surfaces and the rare photocatalytic properties of titanium dioxide provide a rationale for the study of systems comprising both. Such systems can be realized in the form of SAMs grown on TiO_2_ or, in a complementary manner, as TiO_2_ grown on SAMs. Accordingly, the current status of knowledge regarding SAMs on TiO_2_ is described. Photocatalytic phenomena that are of specific relevance to SAMs, such as remote degradation, and cases where SAMs were used to study photocatalytic phenomena, are discussed as well. Mastering of micro-patterning is a key issue en route to a successful assimilation of a variety of titanium dioxide based devices. Accordingly, particular attention is given to the description of a variety of methods and techniques aimed at utilizing the photocatalytic properties of titanium dioxide for patterning. Reports on a variety of applications are discussed. These examples, representing the areas of photovoltaics, microelectronics, microelectromechanics, photocatalysis, corrosion prevention and even biomedicine should be regarded as appetizers paving the way for further studies to be performed.

## Introduction

Photocatalytic degradation of pollutants is attracting increasing attention. In this context, anatase-phase titanium dioxide is regarded as the photocatalyst of choice, due to its low cost, nontoxicity, and relatively high efficiency, which make it suitable not only for air and water decontamination [1–2] but also for self-cleaning applications [3]. The general scheme for the photocatalytic destruction of organics involves the excitation of this semiconductor by irradiation with suprabandgap photons and migration of the electron–hole pairs to the surface of the photocatalyst, where the holes are trapped by H_2_O or OH^–^ adsorbed at the surface, thus forming hydroxyl radicals. In parallel, the electrons reduce adsorbed oxygen [4] to form superoxide radicals. The first step in the photocatalytic degradation of most organic compounds is an oxidative attack by the hydroxyl radicals, which eventually, following secondary reactions, gives stable molecules such as CO_2_ and water [5–6]. Nevertheless, it was shown that some halo-organics [7–8] and highly toxic heavy-metal ions such as Cr(VI) [9–10] could be degraded reductively by photoinduced electrons. Langmuir–Hinshelwood type kinetics is often observed both in the liquid phase and in the gas phase, suggesting, albeit not proving [11–12] the need for adsorption as a prerequisite for photocatalysis.

Self-assembled monolayers (SAMs) being chemisorbed in an ordered manner on surfaces such as metals (Au, Ag), oxides (SiO_2_, Al_2_O_3_, TiO_2_) and semiconductors (Si, GaN, InP, InGaAs) provide a unique way to alter the properties of a surface at will. This ability may be manifested through a variety of phenomena, among which are wetting phenomena (hydrophobicity, hydrophilicity and oleophobicity), electronic phenomena (from affecting band bending and work function, to charge conduction), and, no less important, the ability to form tailored three-dimensional supramolecular arrays by attaching a specific molecule or a particle to an external functional group.

Being adsorbed on the surface of titanium dioxide or in its vicinity, organic self-assembled monolayers may affect the photocatalytic properties of titania as well as be affected by these properties. Likewise, the superhydrophilicity of TiO_2_ known to be induced upon exposure to UV light [13] may affect the chemisorption process of SAMs. This gives rise to diverse phenomena, which can be utilized in many ways, from the study of fundamental issues in TiO_2_ photocatalysis to the growth of supramolecular structures; from serving as a tool for patterning to suggesting means to obtain the selective photocatalytic degradation of highly toxic contaminants. This potential for synergism between self-assembled monolayers and photocatalytic titanium dioxide is the subject of the following review, whose aim is to bring the prospects and obstacles of this combination to the attention of the scientific community. It should be noted that for obvious reasons this manuscript does not cover devices where the titanium dioxide serves to accept photoinduced electrons from sensitizers that cannot be strictly considered as SAMs, i.e., most types of dye-sensitized solar cells (DSSCs).

## Review

### Self-assembled monolayers chemisorbed on TiO_2_

Both TiO_2_ and SiO_2_ are oxides capable of forming surface hydroxyls, and therefore one could imagine that SAMs on titania may resemble SAMs on silica. This similarity is expected to be manifested primarily by the type of head groups that connect between the surface and the organic tails. Indeed, head groups such as chlorosilanes (R*_n_*SiCl_4–_*_n_* with *n* = 1,2,3), alkoxysilanes (R*_n_*Si(OR')_4–_*_n_* with *n* = 1,2,3), carboxylic acids and isocyanates (–N=C=O) are common on both substrates. The fact that the Si–O bond length in silica (1.5–1.7 Å depending on the crystalline form) is similar to that of the Ti–O bond length in titania (1.9 Å) may suggest similar compactness. On the other hand, the difference between the electronegativity of Ti to that of Si, (1.54 and 1.90 by Pauling’s scale, respectively) which affects the polarity of the M–O bond of the oxide, the point of zero charge of the oxide, and the number of OH sites on the surface are expected to influence the tendency of these two oxides to form SAMs and the structure and stability of the formed SAMs.

Chlorosilanes and alkoxysilanes SAMs are characterized by hydrolysis–condensation reactions leading to the formation of M–O–Si bonds where M is in this context is Si or Ti. The chemical anchoring of the alkylsilanes to TiO_2_ is characterized by several changes in the FTIR spectrum, namely shifting of the 1091 cm^−1^ band found in neat TiO_2_ (bending vibration of Ti–OH) to lower wavenumbers (ca. 1000 cm^−1^) due to the formation of Ti–O-Si bonds, and the disappearance of the in-plane-bending vibration of surface O–H at 1402 cm^−1^ [14].

Generally speaking, a comprehensive comparison between SAMs on silica and SAMs on titanium dioxide is somehow problematic as the latter were by far less-extensively studied than the former. It is commonly claimed that silanes capable of cross linking (i.e., having at least three leaving groups) grow by an islandlike growth mechanism, whereas SAMs that are not capable of cross linking grow by a uniform growth mechanism [15]. While this is well-established for SAMs on Si, results for organosilanes SAMs on titanium dioxide are much more ambiguous. The lower electronegativity of titanium suggests that the condensation reaction is faster than on SiO_2_, and as a consequence the grafting of octadecyl trichlorosilane (OTS) on TiO_2_ is faster [16]. To some extent this is related to the known ability of Ti(OR)_4_ to catalyze silanol condensation in TiO_2_–SiO_2_ sol–gel systems. Since island formation of OTS molecules requires lateral mobility, which may be hindered if the grafting is too strong, one may expect the OTS islands on TiO_2_ to be smaller than on SiO_2_.

There are several indications (most of them based on the FTIR signal of the C–H stretch envelope) that the amount of chemisorbed trichlorosilane molecules is higher in TiO_2_ than in SiO_2_, possibly due to the presence of surplus water [17] or, in the case of TiO_2_ films consisting of sintered nanocrystalline TiO_2_, due to a difference between the geometrical area and the true area [18]. Conversely, the density of a protein, immobilized on a substrate through an alkylsilane SAM having a terminal amine, was observed to be lower on a TiO_2_ substrate than on a SiO_2_ substrate [19]. Here, it was claimed by the authors that the more ionic character of the Ti–O bond may require higher energies to form Ti–OH groups, leading to lower density of surface hydroxyls unless an extended exposure to O_2_ plasma in the presence of water vapor is performed.

The formation of OTS monolayers on titanium dioxide was studied in structures consisting of well-defined microdomains of TiO_2_ and noble metals such as gold and platinum. It was found that monolayers chemisorbed in the presence of the metallic micro-islands were denser than monolayers chemisorbed on TiO_2_ substrates that had no metallic islands. Results were explained in terms of charging effects [18]. That charging of the substrate may affect the chemisorption of organosiloxane monolayers can be deduced also from a comparison between SAMs on SiO_2_, on mica and on mica coated with ultrathin layers of SiO_2_. Here, it was found that the adsorption rate decreased with the width of the silica overlayer, and this result was explained by the increased shadowing of an electrostatic interaction between the negatively charged mica surface and the polar head group of the adsorbed molecules [20].

The effect of raising the temperature may be manifested in SAMs through disordering (formation of “kink” configurations), detachment of the molecules, or burning. FTIR studies of temperature effects on a variety of organosilane SAMs on TiO_2_ found that all the organosilane SAMs exhibited good thermal and oxidative stability, with no mass loss below 200 °C [15], as is known also for organosilanes on silicon [21]. A different study on in-air pyrolization of thioacetate-terminated (trichlorosilyl) hexadecane on SiO_2_ and TiO_2_ did not reveal any substrate effect on the onset of burning and on the temperature dependence of the process [22].

Data on contact-angle comparisons between organosilanes on silica and on titania is quite scarce. In this respect, contact-angle measurements of CVD-made tetrafunctional cyclic siloxane monolayers (1,3,5,7-tetramethylcyclotetrasiloxane (C_4_H_16_O_4_Si_4_)) did not reveal much of a difference between SAMs on oxidized titanium versus SAMs on oxidized aluminum [23]. In both cases, the water contact angle was found to be 103° when the CVD process took place at 80 °C, and 163° when the process took place at 180 °C. The *n*-hexadecane contact angles were also the same for both substrates, i.e., 32° and 0° for monolayers grown at 80 °C and 180 °C, respectively. The fact that the contact angles on these very short SAMs (0.5 nm in thickness) revealed a lack of sensitivity to the type of substrate suggests (albeit not proves) that a similar situation may prevail also with SAMs having long alkyl chains, whose outer groups are located far from the substrate and in which multiple intermolecular van-der-Waals (VdW) interactions play a larger role.

The high solubility of polysiloxanes in CO_2_ led researchers to study the silanization of titanium dioxide under supercritical conditions. It was found that despite a tendency to form a disordered, three-dimensional silanized structure [24], a monolayer with a very low degree of vertical polycondensation can be obtained at pressures above 10.0–12.5 MPa [25]. Such monolayers have a relatively lower grafting density with respect to chemisorption by conventional methods (2.8–3.0 molecules per nm^2^ versus 4.3–4.8 molecules per nm^2^).

It is worth mentioning that a study on organosilane monolayers formed on the surfaces of zirconia and titania (anatase and rutile), by a gas–phase process employing organosilicon hydrides, found that the effect of the underlying substrate on the adsorption of nitrogen on the SAMs was insignificant [26]. Here, the heat of adsorption of the nitrogen molecules was found to increase as the grafting density of the SAMs was decreased from 4.23 groups/nm^2^ for C_18_H_37_SiH_3_ to 2.75 groups/nm^2^ for H_3_Si(CH_2_)_8_SiH_3_.

Unlike organosilane SAMs, whose tendency to form on TiO_2_ and SiO_2_ is quite similar, SAMs having phosphonic acid as their connecting head group are not formed on silicon dioxide but are formed easily from aqueous solutions on TiO_2_, Al_2_O_3_, Ta_2_O_5_ and Nb_2_O_5_ [27].

FTIR measurements of self-assembled alkanephosphate monolayers revealed a clear shift in the symmetric and antisymmetric methylene stretching bands toward lower wavenumbers with increasing adsorption time, indicating a change from a disordered conformation to a well-ordered structure [28]. The observation of a disorder–order change for alkanephosphate SAMs on TiO_2_ supported the validity of the uniform growth mechanism, i.e., strong chemisorption of single molecules that once chemisorbed are incapable of surface diffusion. The dichroic ratio of the methylene antisymmetric stretching band, defined as the intensity ratio of the band in the two polarizations (*A*_s_/*A*_p_), was found to increase with adsorption time and to level off at a ratio of 1.3, further supporting the uniform growth mechanism. The results of the dichroic ratio for the well-packed monolayer were analyzed under the assumption of uniaxial orientation, yielding a tilt angle of the alkyl chains of 21° relative to the surface normal. It should be noted that the uniform growth mechanism is considered to be typical for molecules that do not cross link (such as Si(CH_3_)_2_-Cl for example) [15], hence it may imply that this was the case also with the alkanephosphates.

**Figure 1:**
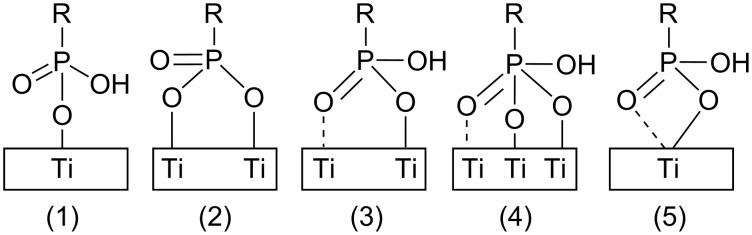
Schematic representation of binding modes between phosphonic acid SAMs and titanium dioxide (1) monodentate, (2) and (3) bridging bidentate, (4) bridging tridentate, (5) chelating bidentate (adapted from [29]).

The binding of self-assembled monolayers of ^17^O-enriched phosphonic acids chemisorbed on titanium dioxide was studied by high-field NMR [29]. The presence of P–O–Ti, P=O, and P–OH indicated that mono-, bi- and tridentate surface phophonate units can be present in these monolayers (Figure 1). The relative contribution of each form was found to vary according to the tail group, namely the relative contribution of P–O–Ti, P=O and P–OH was found to be different for PhPO_3_H_2_/TiO_2_ and C_12_H_25_PO_3_H_2_/TiO_2_. Unfortunately, the lack of uniqueness in the assignment of the relative contributions to the three forms of anchoring prevented calculation of the relative role of each type of anchoring. At any case, the chemical shift of the P–O–Ti sites was found to be consistent with bridging modes, negating the possibility of anchoring through chelating modes.

A slightly different view of the binding between *n*-monoalkanephosphate SAMs and TiO_2_ was presented by Chen et al. who claimed, based on XPS measurements, that this type of SAM can be bonded to the TiO_2_ surface by way of both monodentate and bidentate coordination [30]. Accordingly, it was claimed that the monodentate and the bidentate of adjacent phosphate headgroups are linked by intermolecular hydrogen bonding.

An interesting phenomenon was found with SAMs connected to the TiO_2_ surface through a carboxylic acid group. Here, doping TiO_2_ nanoparticles with Co^2+^ at high concentrations (up to 23%), where the Co^2+^ replaces Ti^4+^ by substitution, was found to significantly improve the solubility and dispersibility of the nanocrystals in aprotic solvents, upon coating with thin films of oleic acid (CH_3_(CH_2_)_7_CH=CH(CH_2_)_7_COOH) [31]. The aggregation on undoped particles was explained by the oleic acid forming a bilayer, with the carboxylic groups located at the solvent interface. In contrast, in doped particles, a monolayer exposing its hydrophobic functional groups to the aprotic solvents is formed, thus stabilizing the dispersion. This dependency in the formation of the thin layer on the doping was claimed to be related to the packing of the first layer. On doped nanoparticles the formed monolayer was denser than on undoped examples, thus preventing the interpenetration of hydrophobic chains that could have formed the bilayer structure.

Another functional headgroup used for the formation of SAMs on titanium dioxide is isocyanate (CH_3_(CH_2_)*_n_*N=C=O), which forms a relatively weak carbamate linkage with the surface [32]. Here, water contact-angle hysteresis for the SAM-covered TiO_2_ surfaces were found to be larger than that observed for the SAM-covered SiO_2_ surface, suggesting that alkyl isocyanate SAMs on TiO_2_ were more disordered and/or were less densely packed compared with alkyl isocyanate SAMs on SiO_2_. Similar to other SAMs on many substrates, the longer the alkyl chains were, the more stable were the SAMs, by virtue of a larger number of VdW interactions.

### TiO_2_ grown on SAMs

There are quite a large number of manuscripts describing the growth of titanium dioxide on top of SAMs. The content of most of these publications is of little relevance to this mini-review, since in most cases the photocatalytic properties of the grown TiO_2_ were not demonstrated. This lack of documented activity is at least partially related to the fact that in most cases the grown titanium dioxide was not in the photocatalytic anatase phase but rather it was amorphous. This amorphous phase can be transformed to anatase; however, it requires temperatures no less than 300 °C, which are expected to severely damage the underlying organic SAM.

Generally speaking, there are three main methods for growing titanium dioxide particles and films on SAMs: Liquid-phase deposition (LPD), atomic-layer deposition, and sol–gel. Within the context of growing TiO_2_ on SAMs, the LPD method is probably the most popular. It employs a solution containing TiF_6_^2^**^−^** anions together with boric acid. The fluoride ligand serves to slow down the hydrolysis of the titanium fluoride complex (Equation 1), enabling the deposition of the formed titanium dioxide on the SAMs, whereas the borate ions act to scavenge the fluoride ions formed during hydrolysis according to Equation 2.

[1]
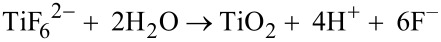


[2]
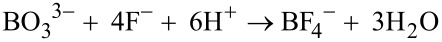


As described below, the phase of the titanium dioxide obtained by this technique depends heavily on the substrate (namely the outer group of the SAM, the pH and the temperature).

Sulfonate (–SO_3_H)-terminated SAMs can be used as substrates onto which nanoparticles and thin films of titanium dioxide can be deposited by an aqueous Ti(IV) route [33]. Here, the sulfonate group provides high local acidity and negative charge even at low pH, thus promoting the hydrolysis and surface attachment of solvated titanium-containing species. It is noteworthy that the fast growth rate on sulfonic-terminated SAMs was also found when the titanium dioxide was grown from a solution containing titanium sulfate and hydrogen peroxide [34]. Obtaining SAMs with sulfonate outer groups is not trivial. It is usually done either by reacting chemisorbed SAMs having a thioacetate terminal group [33] or by reacting terminating thiol groups with H_2_O_2_ in acetic acid [35–36].

By choosing sulfonate-terminated SAMs with long alkyl chains (or a sulfonate-capped polyelectrolyte multilayer) and by careful manipulation of the solution parameters, an anatase phase can be obtained with this method, without the need for high-temperature treatment. The same LPD conditions, but with a silicon substrate instead of a sulfonate-terminated SAM, yielded an amorphous film, demonstrating the importance of the substrate [37]. Low-temperature growth of anatase by LPD was also demonstrated with amine-terminated SAMs, taking advantage of the fact that at pH 2.8, the substrate was charged positively, whereas the TiO_2_ precursor and the nucleated TiO_2_ were charged negatively, as confirmed by ζ potential measurements [38]. At the time, this route was considered to be of large importance for photocatalysis, since (in the case of the aqueous route) it provided a way to form the photoactive anatase phase at temperatures lower than 100 °C, compared with 300–350 °C required in the sol–gel process, or with 170–240 °C required in the TiCl_4_ process performed under vacuum [39]. Meanwhile, other low-temperature processes for producing anatase, such as the titanyl sulfate route [40], have been developed.

Apart from the sulfonate terminated SAMs and the amine-terminated SAMs, the LPD method was used also for the growth of titanium dioxide of unknown phase on SAMs (octadecyltrichlorosilane, phenyltrichlorosilane, vinyltrichlorosilane and *p*-tolyltrichlorosilane) that had been partially oxidized to yield –OH termination. The importance of this work was not in the growth itself, but rather in the fact that the underlying SAMs served as linkers to a polymeric substrate consisting of (aminopropyl)triethoxysilane grafted poly(ethylene terephthalate) [41].

The use of sol–gel methods, utilizing titanium alkoxides as TiO_2_ precursors in an alcoholic medium is a well-known technique for forming TiO_2_ (albeit not anatase) on solid substrates. The method was applied for the growth of titanium dioxide on –CH_3_- [42–43], –OH- [44], and –COOH-terminated SAMs [45]. In the last work the authors compared a two-steps method, in which a HS–(CH_2_)_10_–COOH monolayer was first adsorbed on gold and then exposed to an ethanolic TiO_2_ colloid solution, and a one-step process in which an ethanolic colloid of TiO_2_ nanocrystallites was prepared by the sol–gel method in the presence of the functionalized thiols prior to adsorption onto the gold surface. It was found that the one-step process yielded a lower coverage of the TiO_2_ nanoparticles due to the formation of HS–(CH_2_)_10_–COOH spacers connected to the titania nanoparticles. Similarly, Langmuir–Blodgett films of 1,12-dodecane dicarboxylic acid were used to connect a monolayer of TiO_2_ spheres to silicon and glass substrates, upon performing a dehydration–condensation reaction between the carboxyl groups of the dicarboxylic acid and the surface hydroxyl groups on both the substrate and the ceramic spheres [46]. It was claimed that the flexibility of the alkyl chains in the LB film plays a role in improving the capturing of the spheres.

Atomic-layer deposition (ALD) is a gas–phase thin-film deposition method employing self-terminating surface reactions, leading to a linear correlation between the thickness of the layer and the number of deposition cycles. Mixed SAMs with different ratios of –OH- and –CH_3_-terminated groups were used to control the surface energy and, as a result, to affect the growth of TiO_2_ by ALD from titanium isopropoxide and water [47]. Here, two-dimensional growth was observed on SAM-coated substrates with high surface energy, whereas a three-dimensional growth mode was found on SAM-coated substrates with low surface energy. The high affinity between OH groups and the titania precursor was later utilized for the growth of patterned domains of titania on patterned OH-terminated alkanethiolate monolayers on gold [48].

### SAMs as a means for studying photocatalysis

The fact that SAMs are adsorbed irreversibly (or almost irreversibly) on the surface of titanium dioxide makes them a valuable tool for studying fundamental phenomena in photocatalysis, as they provide a way to decouple adsorption and reaction. In this manner, SAMs were utilized to study the so-called “remote degradation” effect, namely the ability to photocatalytically decompose molecules that are located away from the TiO_2_ surface. Here, a cross-linked SAM of OTS was chemisorbed on well-defined structures comprising alternating microstripes of titania and oxidized silicon of equal width. Upon exposure to UV light, complete mineralization of the OTS located on both types of substrates was observed, even in stripes as wide as 40 µm. The measured degradation kinetics on the TiO_2_–Si micropatterned structures was fitted by a bi-exponential fit with two distinct apparent activation energies. Accordingly, it was suggested that oxidizing species leave the titanium dioxide domains to photocatalytically degrade molecules anchored on the remote silicon domains [49]. The remote degradation of OTS on the native oxide of silicon in a structure consisting of alternating stripes of silicon and titania was confirmed later by AFM measurements [16]. Here, XPS measurements showed that, upon complete degradation, the siloxane headgroups remain on the TiO_2_ surface.

Unlike OTS located on silicon in the vicinity of TiO_2_, monolayers of ODT (CH_3_(CH_2_)_17_SH), attached to similar stripes made of gold or platinum located in the vicinity of TiO_2_, were found to be quite resistant to remote degradation [50–51]. This stability was explained by the high cross section for the reaction between OH radicals and gold relative to that with silica, and was the basis for the development of photocatalysts having specificity, which utilized the “adsorb & shuttle” concept.

In a different study, octyltrichlorosilane (OCTS) SAMs chemisorbed on TiO_2_ microelectrodes in an interdigitated TiO_2_/Pt array were used to study the performance of an electrophotocatalytic cell as a function of applied bias [52]. The applied bias acted to push photogenerated holes to the external surface of the TiO_2_ layer while pulling the photogenerated electrons to the platinum electrons, thus limiting the recombination rate. Indeed, the degradation rate constant was found to increase as the positive bias on the photocatalyst was raised up to 0.4–0.6 V. Unexpectedly, as the bias was increased above that level, not only did the degradation rate not increase, but in fact the oxidation rate of the SAM began to decrease.

The use of chemisorbed monolayers was crucial for understanding these results. If this phenomenon of counter-productive bias had been measured with a liquid-phase contaminant, one could have claimed that the observed decrease in the rate was due to a significant decrease in the adsorption rate of the target molecule. Here, the fact that the OCTS molecules were chemically and irreversibly attached to the TiO_2_ electrodes suggested that there had to be another reason. Superoxide radicals, though by themselves ineffective agents for initiating the degradation, may play an important role in the secondary stages of many photocatalytic processes. Hence, a possible explanation could be a shortage of superoxide radicals, as these were formed at the reduction sites, namely at the platinum electrodes. Similar conclusions were drawn also from experiments in air upon studying a nonbiased system consisting of micrometer-size domains of TiO_2_, onto which OTS was chemisorbed in close contact with micrometer-size domains made of gold and platinum [53]. The effect was found to depend on the size of the metallic domain, as well as on the humidity and on the type of metal. Overall, it can be concluded that the use of SAMs to study photocatalysis provided a unique tool to elucidate the role that superoxides may play in photocatalysis, a role that is quite often overlooked.

It is noteworthy that this discussion of the photocatalytic degradation of SAMs is based on the presumption of indirect oxidation, i.e., the transformation of the oxidative power of the photoinduced holes into oxygen-containing species, such as OH radicals. While this indirect oxidation is by all means the prevailing degradation mechanism in almost all organic species physisorbed on the surface of the photocatalyst, the situation can be different when the organic molecules are covalently bound to the surface. Indeed, the photocatalytic degradation of octadecyltrimethoxy silane (ODTMS) SAM on n-type GaN was attributed to a direct mechanism involving electron transfer from the HOMO level of the ODTMS to the valence band of the excited GaN [54]. As a consequence of this direct mechanism in gallium nitride, no remote degradation effects were observed on this photocatalyst. In contrast, the observation of remote degradation on TiO_2_ indicates that indirect oxidation is the dominant mechanism on titanium dioxide. This conclusion is supported also by the fact that the rate of degradation of alkylphosphonic acid SAMs was found to correlate inversely with the ability of oxygen-containing species to reach the surface by penetrating in between the chains of the monolayer [55].

### Surface patterning

Patterning of surfaces is one of the key issues in many applications involving SAMs. Generally speaking, patterning is manifested by the production of at least two types of surfaces having predesigned geometries that differ in at least one specific property. These properties can be chemical, electronic, optic, acoustic, etc. One of the most popular contrast mechanisms is the contrast between hydrophilic and hydrophobic surfaces, in particular since it can be utilized for selective deposition or growth of a large variety of materials.

The photocatalytic properties of titanium dioxide, enabling it to oxidize SAMs under the relatively weak intensity of UV light, together with the superhydrophilic nature of TiO_2_ upon exposure to that light and its mechanical and optical characteristics make titanium dioxide a very interesting material for patterning. Indeed, scientific manuscripts on patterning of surfaces are the majority among those articles discussing both titanium dioxide and self-assembled monolayers.

In the context of SAMs, there are a large number of ways in which patterning can be manifested. Partial coverage of the substrate by SAMs, coverage of the surface with more than one type of SAM and selective deposition of materials on prepatterned SAMs, is only a partial list of examples; our discussion of the patterning techniques is organized accordingly, addressing namely the patterning of SAMs on TiO_2_ and the patterning of TiO_2_ on SAMs by selective growth.

### Patterning of SAMs on TiO_2_

**Figure 2:**
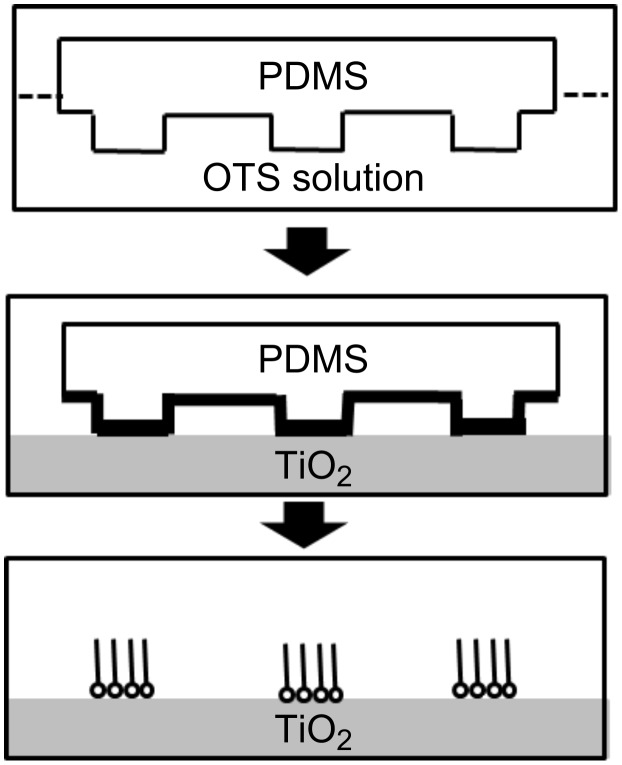
Patterning of SAMs on titanium dioxide by the microcontact printing method.

Patterning of SAMs on TiO_2_ can be obtained by both photocatalytic and nonphotocatalytic routes. Among the nonphotocatalytic methods is microcontact printing (Figure 2) [56], in which SAMs are transferred from stamps of a polymer (for example poly(dimethylsiloxane) (PDMS)) onto oxide substrates upon contact between the stamps and the substrate. Other so-called “soft-lithography” methods (replica molding, microtransfer molding, micromolding in capillaries, and solvent-assisted micromolding) may work as well [57]. For example, colloidal lithography was used to create gold nanopits on a TiO_2_ matrix, onto which methyl-terminated alkanethiol SAMs were chemisorbed [58].

**Figure 3:**
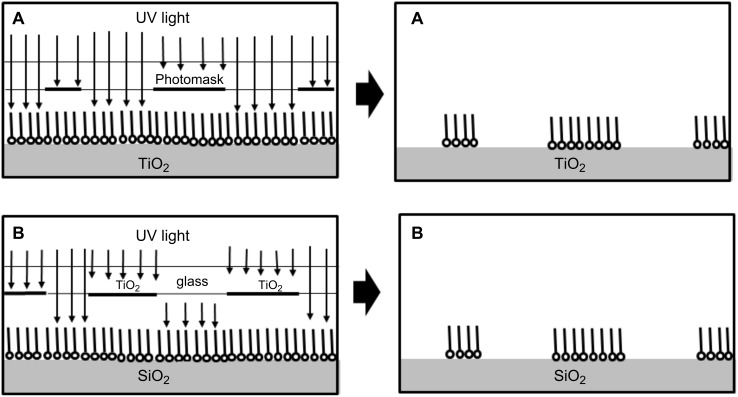
Photocatalytic patterning of SAMs. (A) SAMs on TiO_2_ (B) SAMs on inert substrates.

While photochemical patterning of SAMs on a variety of substrates without the use of designated photoresists is possible under exposure to 185 nm light [59], it is limited to specific functional groups, under constrained environments. In contrast, SAMs located on titanium dioxide can be patterned quite easily by photocatalysis (Figure 3A). There is no need for a photoresist, and a standard patterning mask can be used, or otherwise one may imprint water-based ink patterns on the SAMs, which will prevent the photocatalytic degradation of the shadowed area [60]. Instead of exposure through a mask, one may “write” with a well-collimated beam of UV radiation, for example by using an UV laser, or by near-field optical microscope coupled to an UV laser [55].

An interesting (but alas quite cumbersome) way to obtain patterned surfaces with hydrophilic–hydrophobic contrast is to form a prepatterned area comprising TiO_2_ and another oxide by conventional lithography, and then to attach a hydrophobic SAM to the whole area. The hydrophobic–hydrophilic patterning is then obtained photocatalytically by exposure of the entire area to UV light, thus, degrading the SAM from the TiO_2_ domains. This approach was demonstrated with CuO domains prepared by oxidation of Cu that had been deposited by electroless deposition on silver [61]. The silver was deposited on a titania film by photocatalytic reduction. Thus, in this case the photocatalytic properties of titanium dioxide were exploited twice, i.e., for the deposition of the silver domains and for the degradation of the SAMs chemisorbed on the photocatalyst domain. It is noteworthy that the hydrophobic spots were relatively large (0.5 mm in diameter), such that remote degradation effects were less acute for this system.

One of the problems associated with the formation of hydrophobic–hydrophilic contrast patterns comprising a hydrophobic SAM on TiO_2_ and superhydrophilic titanium dioxide is the loss in contrast over time, which is due to eventual contamination of the TiO_2_ surface upon adsorption of organic molecules from the air. Exposure to UV light may degrade these molecules, thus, restoring superhydrophilicity; however, it might also degrade the organic SAM and therefore cannot be used to solve the problem of contrast loss. A novel approach for the construction of a renewable superhydrophobic–superhydrophilic surface was presented by Nishimoto et al. [62]. The approach is based on through-mask photocatalytic patterning of hydrophobic SAM on TiO_2_, followed by deposition of boehmite (AlOOH·*n*H_2_O) on the exposed TiO_2_ domains. Then, a heat-treatment step converted the boehmite into Al_2_O_3_, while oxidizing the SAMs, forming a patterned TiO_2_–boehmite surface. A hydrophobic SAM was then attached to both types of domains, which then went through a second step of exposure to UV light. At the end of the process a negative image of the first-step surface was obtained, consisting of superhydrophilic TiO_2_ domains and superhydrophobic domains anchored to alumina. In that way, the restoration of hydrophilic contrast by exposure to UV was expected not to take its toll on the hydrophobic SAMs. With respect to remote degradation, the fact that the inert substrate here is alumina and not silica may assist to preserve the SAMs, as can be inferred from a comparison of the remote degradation effects of SAMs on silica to those of SAMs on alumina [51].

Remote degradation effects are not necessarily destructive when it comes to the patterning of SAMs. In fact, they can be utilized to pattern SAMs on inert surfaces (Figure 3B). The technique was demonstrated by Lee and Sung, who used a quartz mask containing patterned TiO_2_ in order to pattern an octadecylsiloxane SAM on silicon [63]. Once patterned, ultra-thin layers of ZrO_2_ were deposited by atomic-layer deposition on the exposed parts of the silicon substrate. The reported spatial resolution was striking: The nominal width of ZrO_2_ lines and SAM-coated Si lines was approximately 0.5 µm. This relatively high resolution should be attributed to the intimate contact between the TiO_2_ mask and the SAM-coated silicon as well as to the short exposure time, which minimized the blurring.

Another example of photocatalytic patterning of SAMs on inert substrates is the patterning of perfluorodecanethiol SAM on gold by a through-mask back exposure of thin films of titania located at a distance of 12.5 µm from the SAM-coated gold [64]. The quality of the patterned surface was examined by immobilizing a fluorescent dye on the oxidized regions of the patterned gold surface. The same method was used to pattern enzymes on a gold surface, by the attachment of fluorescein isothiocyanate labeled peroxidase (FITC-POD) onto the hydrophilic regions. Unfortunately, no details were given regarding the thickness of the TiO_2_ layer and the wavelength. Such details could be of high importance for analyzing the significance of the data in this back-exposure configuration.

Photocatalytic lithography by remote degradation was also demonstrated by the formation of grayscale gradients in thiolated SAMs anchored to gold located as far as 60 µm from a TiO_2_ thin film on quartz, back-irradiated through a mask [65]. The gaps in the thiolated SAM were then filled with 11-mercapto-1-undecanol or with 1*H*,1*H*,2*H*,2*H*-perfluorodecanethiol. The flux during irradiation was quite high (17 mW/cm^2^). The apparent contradiction between this study and works that reported the high stability of SAMs on gold towards remote degradation [50] may be explained by the different position of the SAMs relative to the source of the oxidizing species and the high UV flux in the back-irradiation experiments.

An interesting, inexpensive way to use photocatalysis for the formation of patterns having hydrophobic–hydrophilic contrast was presented by Bai et al. [66]. Here, TiO_2_ particles in solution were used to pattern an OTS monolayer on mica sheets, the size of the islands and concentration being affected by the UV flux impinging on the surface, as evidenced by AFM and wettability measurements.

The phenomenon of remote degradation raises a question regarding the fidelity of patterns obtained by through-mask exposure techniques. Indeed, exposure of OTS-coated TiO_2_ to 254 nm light through a quartz mask covered with chromium stripes (40 µm in width and distance) caused a complete degradation of the alkyl chains, including those in the “dark” regions [67]. This does not necessarily contradict the reports on patterning presented above, since analysis of the kinetics revealed that the degradation rate in the exposed areas was 2–20 times faster than in the dark areas, and hence, obtaining a reasonable contrast is still possible. However, it definitively demonstrated that patterning can be very sensitive to overexposure, and that in terms of contrast, the best resolution that can be achieved with photocatalytic patterning is expected to be no better than a few microns. Moreover, if one accepts the notion that the dominant mechanism of remote degradation is photoinduced homolysis of photocatalytically formed hydrogen peroxide, then an important outcome is that structures that are patterned by exposure to 365 nm light may be sharper than structures patterned by 254 nm light. This conclusion, which seems contradictory to conventional wisdom, stems from the fact that the quantum efficiency of the generation of OH-radicals by photohomolysis of H_2_O_2_ with 365 nm photons is 110 times smaller than that with 254 nm photons [68].

### Patterning of TiO_2_ by selective growth on SAMs

Probably the most popular way by which SAMs have been used as a means to obtain patterned TiO_2_ films is through site-selective deposition (SSD) of the oxide on prepatterned SAMs [69]. The concept here is to pattern SAMs on substrates, either by complete removal or by site-specific tailoring of the outer groups, thus forming areas with high tendency for titania growth, coexisting with domains onto which titania will not grow. It should be pointed out that the SSD technique is not limited to the deposition of titanium dioxide and was utilized for patterned growth of other oxides such as In_2_O_3_ [70], Ta_2_O_5_, SnO_2_ and SrTiO_3_ [69].

**Figure 4:**
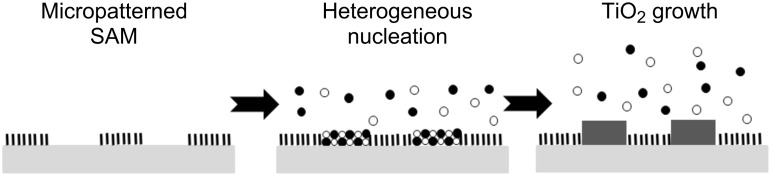
Patterning of SAM on a substrate followed by selective growth directly on the substrate.

The most popular means for selective growth is direct site-selective deposition (Figure 4), based on patterning of SAMs on a substrate (either by exposure to 185 nm light or by conventional photolithography), followed by nucleation and growth of TiO_2_ on areas that have been depleted of the SAMs. As an example, one may mention the patterning of OTS into methyl-terminated regions and silanol-terminated regions, onto which amorphous titanium dioxide formed from titanium dichloride diethoxide (TDD) was deposited either from the liquid phase (D-40) [71] or from the gas phase [72]. The latter was reported to yield higher quality films due to a lack of bulk nucleation. In a later study a comparison was made between three types of precursors, namely TDD, titanium tetrachloride (TC) and titanium tetraethoxide (TE), acting on deep UV-exposed OTS and PTCS (phenyltrichlorosilane) [73]. Quite surprisingly, it was found that the contrast in the patterns of the grown oxide depended on the type of precursor. While TC or TDD formed TiO_2_ on the hydrophilic silanol groups but not on the hydrophobic methyl groups of OTS, TE induced TiO_2_ growth on both types of substrates without any preference. Regardless of the precursor, the obtained TiO_2_ films were amorphous. Conversion to anatase took place at 300 °C when TC was the precursor, whereas a temperature of 400 °C was required when TDD or TE were used as precursors. To improve the contrast in SSD growth on a patterned silanol–hydrophobic SAM surface one may use sonication, which has been demonstrated to remove loosely adhered TiO_2_ particles from domains on which deposition was undesirable [74–75].

**Figure 5:**
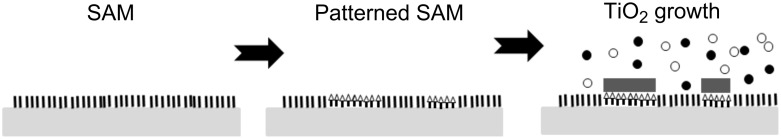
Partial oxidation of SAMs at predesigned locations followed by TiO_2_ growth on the partially oxidized domains.

Exposure to deep UV through a photomask was also used for partial oxidation of SAMs of octadecyltrichlorosilane, phenyltrichlorosilane, vinyltrichlorosilane and *p*-tolyltrichlorosilane on an (aminopropyl)triethoxysilane grafted poly(ethylene terephthalate) surface. Subsequently, TiO_2_ was grown selectively by LPD on the oxidized domains (Figure 5). It was found that SAMs containing aromatic rings were the most suitable for growing titania, producing strongly adhering films with distinct TiO_2_ micropatterns [41]. To improve selectivity, a shielding reagent, reversibly adsorbed on the nonexposed domains of a *p*-tolyltrichlorosilane SAM, was added prior to the TiO_2_ growth step [76]. This shielding reagent, dodecylbenzene sodium sulfonate, was chosen based on its tendency, in aqueous solutions, to attach only to hydrophobically-terminated SAMs, due to its amphiphilic nature. It is noteworthy that bubbling air to constantly replace the LPD solution close to the surface can be quite beneficial for forming crack-free TiO_2_ films, as was demonstrated with patterned SAMs of heptadecafluoro-1,1,2,2-tetrahydrodecyltrichlorosilane (HFDTS) [77]. Another example of the approach portrayed in Figure 2B was the patterned oxidation of thioacetate-(–SCOCH_3_) terminated SAMs to form patterned sulfonate-terminated domains onto which TiO_2_ was grown [78].

A non-photoinduced means to pattern SAMs for selective deposition is microcontact printing (Figure 2). For example, microcontact printing of sulfonic acid terminated SAMs facilitated the growth of patterned TiO_2_ from a solution containing titanium sulfate and hydrogen peroxide [34]. Another example is the transfer of OTS SAM onto silica followed by selective ALD growth of titanium dioxide on the noncoated areas [79]. Likewise, a technique called “edge-transfer lithography” was applied to form lines of titanium dioxide nanoparticles with nanometer-scale resolution [80]. Here, transfer of SAMs from the edges of micron-scale-patterned elastomeric stamps onto silica produced nanometer-scale patterned SAMs, with line widths as small as 60 nm. Such thin lines were obtained by a dewetting and blow-drying process, which trapped silane solution only in the recesses of the molded stamp. In a different work, OTS was deposited by microcontact printing onto both external sides of a nanoporous polycarbonate filter. As a consequence, the ALD growth of titanium dioxide was limited to the inner walls of the polycarbonate filter. In that way, the performance of 100–800 ALD cycles followed by the etching away of the polycarbonate template with chloroform yielded TiO_2_ nanotubes, whose diameter could be predetermined according to the diameter of the pores in the PC filter [81].

**Figure 6:**
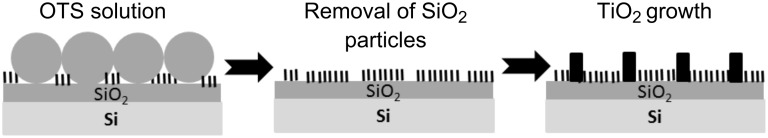
Patterned growth of TiO_2_ by “contact area lithography” (CAL) (after [82]).

Patterning of SAMs en route for selective deposition can be achieved by introducing a physical barrier for the deposition of SAMs, followed by TiO_2_ growth once the barrier is removed. In that manner, coined “contact area lithography” (CAL), round nanoparticles were used to cover a silica surface, thus forming a close-packed structure with a hexagonal pattern of nanometer-sized contact dots (Figure 6). Then, OTS SAMs were deposited everywhere except for on the contact dots, facilitating the ALD growth of nanodisks of TiO_2_ from a titanium tetraisopropoxide precursor [82].

It is noteworthy that the growth rate of anatase on top of SAMs (methyl-terminated or even amino-terminated) is significantly lower than that measured on top of amorphous TiO_2_ underlayer. This was exploited for the growth of a patterned anatase layer on top of amorphous TiO_2_ grown on patterned OH-terminated SAMs [83].

**Figure 7:**
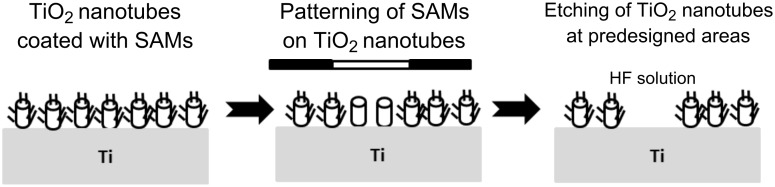
Patterning of a surface containing TiO_2_ nanotubes by localized etching, by using patterned SAMs to protect selected areas (after [84]).

Although SAMs were used to obtain patterned TiO_2_, mainly by directing the deposition of titania, it is possible to pattern TiO_2_ by directing its etching instead of its growth (Figure 7). An interesting example is the patterning of an area made of TiO_2_ nanotubes, formed by anodization of titanium in HF. Here, SAMs of 1*H*,1*H*,2*H*,2*H*-perfluorooctyl-triethoxysilane were chemisorbed on selected areas in the nanotube array and served to selectively protect the nanotubes upon immersion in HF [84].

### Electron transfer in SAMs connected to TiO_2_

Electron transfer through SAMs has been studied quite thoroughly for both organothiolated SAMs on metals and organosilanes on silicon. The appearance of dye-sensitized solar cells [85], based on (disordered) dye molecules attached to the surface of nanoparticulate titanium dioxide, provoked interest also in the charge transport from SAMs to titanium dioxide. In many cases, as detailed below, the SAMs serve as mediators between the sensitizing molecules and the surface, and hence are required to exhibit good conductivity along the molecule, minimal contact resistance, and, no less important, a set of energy levels that can support vectorial charge transfer. Although many of the studies in this area are phenomenological they provide the necessary background required for the development of a variety of microelectronic devices such as solar cells, transistors and capacitors. It is noteworthy that in many publications discussing charge transport between SAMs and titanium dioxide, the characterization of the prepared films is somewhat partial; it is thus very rare to find manuscripts that provide data on the organization and orientation of the adsorbed molecules as well as on their surface concentration.

The finding that C_60_ may transfer electrons to titanium dioxide upon illumination with visible light [86] led to the study of a system in which the C_60_ is anchored to the titanium dioxide through a SAM mediator. The mediator comprised salicylic acid (attached to the TiO_2_ surface through its carboxylate group) connected to a pyrrodine group of a modified C_60_ [87]. An agreement between the measured photocurrent action spectrum and the absorption spectrum of the modified fullerene served as an indication that the photoactive species was the modified fullerene. A photon-to-current conversion efficiency as high as 15% was measured, demonstrating the usefulness of using SAM mediators.

The same concept of using SAMs as mediators was demonstrated in a system comprising quantum dots and self-assembled-monolayer-coated titanium dioxide. Here, cadmium–sulfur–selenium (CdSSe) quantum dots were physically attached to hydrothermally synthesized anatase TiO_2_ nanobelts, onto which SAMs of long chain carboxylic acids, exposing hydrophobic terminating groups, were chemisorbed [88]. An UV-induced compressive force between the nanoparticles and the TiO_2_ nanobelts could be inferred based on Raman spectroscopy. To our understanding, this compressive force may compensate to some extent for the lack of chemical bonding between the quantum dots and the terminating groups of the monolayer, thus, enabling the high photocurrent response measured for this system.

Another study on charge transport between SAMs and TiO_2_ was based on a mixed-monolayer configuration. Here, a self-assembled monolayer of a carotenoid (*trans*-8'-apo-β-caroten-8'-oic acid) was adsorbed on TiO_2_. Long-chain molecules of pheophytin were immobilized in between the long-chain carotenoids by virtue of VdW forces. It was shown that excitation of the pheophytin molecules by 670 nm light was quenched reductively by electron transfer from the carotenoid [89]. It was suggested that the charged pheophytin recovers back to the parent molecule predominantly by injecting an electron into the TiO_2_ conduction band, thus, facilitating the observation of a long-lived carotenoid radical cation. A claim was made that similar paths yielding long-lived charge separation situations may be relevant also in natural photosynthetic systems, and should be considered in the development of dye-sensitized solar cells.

In certain cases, feasibility studies with TiO_2_-containing systems were later implemented in devices that are constructed on other substrates. As an example, a self-assembled monolayer of 5-cyano-2-(butyl-4-phosphonic acid)-3-butylthiophene (CNBTPA) was formed on TiO_2_ by using its phosphonic acid group as a binding group. Once chemisorbed, the monolayer served to attach molecules of α,ω-dicyano substituted β,β'-dibutylquaterthiophene (DCNDBQT) molecules by forming a hydrogen bond between the cyano group of CNBTPA and a hydrogen on the thiophene ring of DCNDBQT, and by forming a hydrogen bond between the cyano group of DCNDBQT and a hydrogen on the thiophene ring of CNBTPA [90]. A quasi-perpendicular structure of the CNBTPA–DCNDBQT layer relative to the TiO_2_ surface was inferred, suggesting optimal orbital overlap between neighboring thiophene rings. The same substituted oligothiophene was then used to form a nanoscopic organic field-effect transistor (OFET), albeit not on TiO_2_ but on Si/SiO_2_ substrate.

### Applications

The study of the ways by which SAMs are attached to titanium dioxide (and, in a complementary manner, ways by which TiO_2_ is grown on SAMs) as well as the study of fundamental phenomena and the developing of patterning techniques, have paved the way for the utilization of systems comprising SAMs and TiO_2_ for a variety of applications. The following section discusses the main applications presented so far.

#### TiO_2_-SAMs in electronic devices

The large dielectric constant *k* of TiO_2_ (25 < *k* < 30) compared with that of silica (*k* = 3.9), as well as its refractory properties, suggest its use in MOSFET technology [91]. In that respect, it is interesting to mention that the dielectric constant of amorphous TiO_2_ grown on patterned OTS from titanic acid (H_2_TiO_3_) [92] was estimated to be 63 at 100 kHz, significantly larger than the reported values of 22 measured for biomimetically deposited amorphous TiO_2_ [93]. This was attributed to the existence of small crystallized particles. A significant drawback in the use of TiO_2_ for microelectronic purposes is the relatively high leakage current (as high as 4.3 × 10^−8^ A cm^−2^ at 1 V and a thickness of 306 nm) and the linear decrease in the dielectric constant as a function of frequency (from *k* = 160 at 1 kHz to *k* = 23 at 1 mHz) [92]. These were explained by the presence of interface states and impurities such as OH^–^ and H_2_O in the film. Still, well-behaved MOSFET transistors with a TiO_2_ gate oxide were demonstrated already in 1997 [94], prior to the full development of patterning technologies. Likewise, a miniature capacitor, made of an oxide–SAM–TiO_2_ sandwiched structure, was presented already in 1998 [95].

The possibility to deposit TiO_2_ on top of sulfonate-terminated SAMs was utilized to form metal–oxide–metal (MOM) heterojunction nanowires by a “bottom-up” approach [96]. Here, Au–TiO_2_–Au nanowires were prepared within nanoholes of anodic aluminum oxide templates. The preparation procedure included the deposition of gold by electroplating, chemisorption of 1,8-octanedithiol (HS–(CH_2_)_8_–SH), oxidation of the terminal thiol groups to form ω-sulfonate groups, deposition of polycrystalline anatase using Ti(OPr)_4_ dissolved in a water–ethanol mixture, and capping of the TiO_2_ with electroplated gold.

Self-assembled monolayers, with their ability to attach both to inorganic and organic materials may have a large potential in hybrid microelectronic systems containing titanium dioxide together with organic components. Along this line, the improvement of the performance of organic field-effect transistors (OFETs) by introducing SAMs was observed with OFETs comprising a titanium gate, dielectric layer of TiO_2_ prepared by anodization, and a poly(triarylamine (PTAA)) layer (alternatively a pentacene layer) that served to form the source and the drain of the transistor [97]. Here, the addition of an OTS layer between the TiO_2_ and the source–drain layer was found to increase the field-effect mobility (calculated in the saturation regime) by two orders of magnitude (with PTAA) or by a factor of 2 (with pentacene).

SAMs may assist in the preparation of hybrid electronic components not only by forming the connection between organic and inorganic layers but also by facilitating self-patterning. In that manner photopatternable SAMs of 1*H*,1*H*,2*H*,2*H*-perfluorododecyltrichlorosilane were used as a template for self-localization of conducting polymers en route to the formation of polymer-based transistors [98]. Here, the source and drain electrodes were formed by spin coating an aqueous solution of poly(3,4-ethylenedioxythiophene) (PEDOT) on TiO_2_/SAM, resulting in dewetting and self-localization of the solution within the exposed domains. The substrates were then further coated with poly(3-hexylthiophene) (P3HT). Conformity of the structure was found to depend heavily on the humidity conditions during exposure, since too high a humidity resulted in remote degradation of nonirradiated areas, which could lead to an excess coverage of PEDOT. For the same reason (prevention of remote degradation) 365 nm light was found to give sharper patterns than 254 nm light.

The use of SAMs on oxidized silicon in order to reduce friction is well documented. In a similar manner, SAMs on titanium dioxide were utilized to simultaneously solve the problems of wear and stiction in microelectromechanical (MEMS) devices. Here, a thin (10 nm) layer of TiO_2_ was coated by the ALD technique onto polysilicon substrates. A SAM of CF_3_(CF_2_)_7_(CH_2_)_2_SiCl_3_ (FDTS) was then chemisorbed on the titania layer [99]. Tribological measurements showed that the static-friction coefficient was dominated by the presence of FDTS as an external layer, as manifested by the fact that the coefficients of FDTS on TiO_2_ and on SiO_2_ were nine times lower than those of SiO_2_ and three times lower than those of TiO_2_. At the same time, wear tests showed that the lifetimes of moving parts were similar to those obtained with polysilicon-coated titanium dioxide, namely 1.5–3.0 times longer than those of noncoated polysilicon.

#### Solar cells

One of the most popular (if not *the* most popular) areas utilizing structures containing SAMs and titanium dioxide, is photovoltaics. In almost all designs these structures are characterized by the SAM serving as a mediator between the photosensitizer and the titanium dioxide acceptor. A variety of photosensitizers have been used: From the conventional ruthenium-based dyes to conductive polymers, C_60_ and inorganic quantum dots. Likewise, a variety of SAMs have been used, with various anchoring groups, including phosphonic acids, silanes, and carboxylic acids. Unfortunately, while the motivation for using SAMs as mediators ensuring vectorial charge transfer is clear, the results obtained so far are still insufficient in terms of cost and efficiency to justify commercial scale production. This does not necessarily mean that the approach of using SAMs as mediators is doomed to fail. On the contrary, analysis shows that this direction draws increasing attention.

Polyaniline (PANI), which has a bandgap of 2.8 eV, compared with 3.2 eV of TiO_2_, was used as a sensitizer, absorbing visible light and transferring photoinduced charge to the titanium dioxide, by virtue of good matching between its LUMO level and the conduction band of TiO_2_. PANI adheres to titanium dioxide by physical adsorption, and thus it was thought that a mediator that forms a strong interaction with both would improve charge transport. Indeed, silane-bearing aniline compound (C_6_H_5_NHC_3_H_6_Si(OMe)_3_) was used to form solvent-free quasi-solid solar cells based on acid-doped polyaniline, but the efficiency was quite modest (0.12%) [100]. Another mediator between PANI and TiO_2_ was aminopropylsilane, resulting in improved thermal stability of PANI and enhanced photocatalytic degradation rate of methyl orange molecules under sunlight, which was attributed to the sensitizing effect of PANI [14].

In a quest to replace expensive dyes in DSSCs, Senadeera et al. used grafted polypyrrole films covalently bonded to self-assembled monolayers of 3-(trimethoxysilyl)propyl methacrylate attached to mesoporous TiO_2_ substrates [101]. Although the overall performance was poor, a comparative study showed that polypyrrole could be used more efficiently as a sensitizer for TiO_2_ when covalently attached through the SAM than it could without the SAM.

The interface between TiO_2_ and poly(3-hexylthiophene)/[6,6,]phenylC_60_ butyric acid methyl ester (P3HT/PCBM), in a based-inverted bulk-heterojunction (BHJ) solar cell, was modified by a series of carboxylic acid functionalized SAMs [102]. The presence of SAMs acted to reduce the contact resistance by passivating the surface trap sites at the TiO_2_ surface, enhancing the electronic coupling between the TiO_2_ and the organic layer, and also improved the growth mode and morphology of the upper organic layer. The largest enhancement was observed with a SAM of C_60_-substituted benzoic acid. Here, the efficiency with the buried SAM layer was 3.8%, compared with 2.8% in the absence of a SAM interlayer.

In two similar systems, two SAMs attached to TiO_2_ through phosphonic acid (2-oligothiophene phosphonic acid and ω-(2-thienyl)alkyl phosphonic acid) were used as interface modifiers on TiO_2_ to increase compatibility with poly(3-hexylthiophene) (P3HT) [103]. The photoluminescence (PL)-quenching efficiency and the short-circuit current density of photovoltaic cells having this configuration were found to increase with the number of thiophene rings and as the alkyl–chain length decreased. Here, a drop in the LUMO level of the interface modifiers increased the photocurrent at the expense of the open-circuit voltage. It should be noted that the observation of correlation between structural parameters in the polymer and its photovoltaic performance is important as it may provide a strategy for stabilizing inorganic particles in the fabrication of high efficiency organic–inorganic photovoltaic devices.

The recent interest in utilizing quantum dots (QDs) for photovoltaics also has its influence on SAMs and titanium dioxide, as more and more cases in which SAMs are used as mediators between QDs and titanium dioxide are published. For example, a self-assembled monolayer of 3-mercaptopropyl-trimethoxysilane was preassembled onto a mesoporous TiO_2_ film to be used as a surface-modified layer to induce the growth of CdSe quantum dots [104]. Here, it was claimed that the terminal thiol groups increased the nucleation and growth rate of CdSe QDs formed by the successive ionic layer adsorption and reaction (SILAR) process. The large uniformity of the CdSe films formed in that way inhibited charge recombination at the electrode–electrolyte interface, and as a consequence, higher efficiency in CdSe-sensitized DSSC solar cells was obtained. In a similar manner, SAMs of mercaptoacetic acid served as substrates for the growth of quantum dots of cadmium sulfide by the same SILAR method [105]. CdS QDs were also produced on SAMs attached to TiO_2_ by a phosphonic acid headgroup [106]. The SILAR procedure here comprised successive cycles consisting of exposure to CdSO_4_, rinsing in DI water, immersion in Na_2_S and a second rinsing in DI water. The solar-cell performance was found to depend on the number of SILAR cycles (it was claimed that above six cycles, the CdS may aggregate or form recombination centers). The efficiency obtained with 3-aminopropyl phosphonic acid (APPA), 3-phosphonopropionic acid (PPA), and 1-butylphosphonic acid (BPA) (0.44%) was as much as three times higher than that measured in the absence of SAMs. Quite surprisingly, no tailgroup dependence was found, suggesting in this case that the CdS nanoparticles were not sitting at the surface of the SAMs, but were rather penetrating into the SAM network such that they resided close to the SAM/TiO_2_ interface.

This part would not be complete without reference to another particular effect of SAMs in a totally different design. This is namely the use of SAMs anchored to titanium dioxide as a means to improve the stability and durability of dye molecules also anchored to TiO_2_. This effect was well demonstrated in the coadsorbption of 1-decylphosphonic acid together with a heteroleptic ruthenium sensitizer that contained two long amphiphilic chains attached to its bipyridine rings (Z-907) [107]. Here, the presence of the SAMs was found to significantly reduce the drop in the open-circuit voltage (from 90 mV to 20 mV) measured following 1000 h of aging at 80 °C. This was achieved without any deleterious effect on the initial performance (approx. 7% efficiency). This enhanced stability was attributed to the ability of the SAM to exclude water molecules from the interface, probably by the formation of a hydrophobic barrier made from the long chained phosphonates interacting with the long amphiphilic chains of the dye.

#### Offset printing

Current offset printing technology is based on anodized aluminum plates patterned, by photosensitive means, into hydrophobic and hydrophilic regions to be wet selectively by oil-based ink and water, respectively. Color printing requires usually 3–4 plates that cannot be recovered. A new type of offset-printing plate that can be reused many times and with a resolution of up to 150 lines per inch was presented recently. This new offset technology is based on photocatalytic patterning of SAM-coated TiO_2_ into superhydrophobic and superhydrophilic regions [108]. Here, the patterning of the SAMs chemisorbed on the TiO_2_-coated plates was performed by using an ink-jet printer to deposit patterned ink, which served as a photomask shielding the SAMs during UV exposure [109].

Loading films or particles of nanoporous titanium dioxide with nanoparticles of silver (for example by photocatalytic deposition upon exposure to UV light) was shown to produce brownish-grey colored surfaces. Illumination of these surfaces by monochromatic visible light changes the color of the Ag–TiO_2_ system to that of the incident light, due to reoxidation, causing the silver nanoparticles that had already absorbed at this specific wavelength to lose their ability to absorb more photons at this same wavelength. This phenomenon, termed “multicolor photochromism” [110] can, in principle, be utilized to form rewritable color papers and paints, or even optical memories. One of the problems preventing this application is the gradual bleaching of the color due to nonpreferential absorption upon exposure to white light. In this context, it was found that modification of the Ag–TiO_2_ films with alkanethiol or fluoroalkanethiol SAMs may help to suppress bleaching, either by preventing the oxidative dissolution of silver or by blocking the electron transfer from silver to oxygen [111]. The mechanism for bleaching suppression is the same as that for coloring suppression, hence reactivation is needed. This reactivation could be obtained by photocatalytic decomposition of the ODT monolayers on the silver by exposure to UV light, and by relying on the remote degradation effect of TiO_2_ as discussed above.

#### SAMs for selective photocatalysis

Heterogeneous photocatalysis, being based on oxidation by hydroxyl radicals, is known to hardly distinguish between different target molecules. Since some contaminants are more toxic than others, and since some contaminants are readily degradable by biological means while others are nonbiodegradable, there is an obvious need to develop selective photocatalysts that will address streams containing multiple contaminants in a manner that would handle preferentially those contaminants that are either highly toxic and/or nonbiodegradable [112].

**Figure 8:**
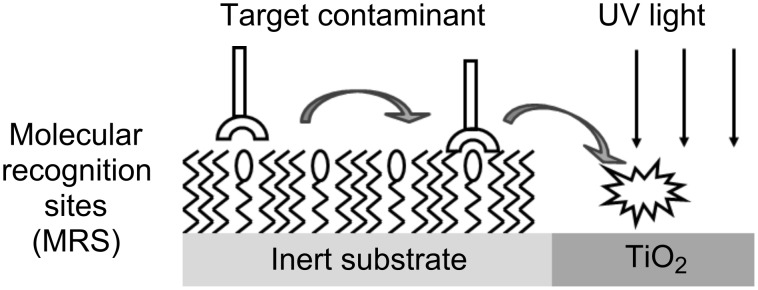
Specific photocatalytic degradation by the “adsorb and shuttle” approach (after [51]).

A few years ago it was proposed that a structure comprising SAMs located in the vicinity of titanium dioxide domains could be used en route to achieve preferential degradation of toxic contaminants. The principle was to use SAMs on inert substrates as molecular recognition platforms able to selectively physisorb specific target molecules. Once physisorbed, the target molecules diffuse from the inert, adsorption sites to the photocatalytic domains, where they are photocatalytically degraded (Figure 8). The feasibility of this approach was first demonstrated by constructing metallic microdomains, onto which self-assembled monolayers of thiolated β-cyclodextrin were chemisorbed. The cavity of the β-cyclodextrin served as a molecular-recognition site for 2-methyl-1,4-naphthoquinone (2MNQ). The measured degradation rate ratio between 2MNQ and benzene was 8.1, compared with 0.8 in the absence of the molecular-recognition sites. As expected, the kinetics was found to depend on the average distance over which the adsorbed 2MNQ had to diffuse in order to get to the photocatalytic domains [113]. The same type of molecular recognition SAM (thiolated cyclodextrin) was found to enhance also the photocatalytic degradation of the dye-stuff Chicago Sky Blue 6, which is a long, symmetric molecule whose chemical structure fits the cyclodextrin cavity [51].

This so-called “adsorb and shuttle” approach was later implemented also by utilizing Cu^2+^ ions attached to SAMs of 1,1-mercaptoundecanate in order to physisorb diisopropyl methyl phosphonate (DIMP), a known simulant for the nerve gas sarin. Once physisorbed, the DIMP molecules diffused to the TiO_2_ domains where they were photocatalytically degraded in a mechanism similar to that observed with TiO_2_ alone, namely, via the formation of acetone as an intermediate product. An enhancement factor of 4–6 relative to bare TiO_2_ was observed [114].

It is noteworthy that the phenomenon of remote degradation discussed above poses a severe limitation to the concept of selective photocatalysis, as the SAMs might be prone to eventual degradation upon exposure to UV light. Placing the molecular recognition sites on thin films of metals such as gold helps to overcome this problem, as it stabilizes the monolayers against remote degradation, nevertheless this solution might be insufficient in powders, where the size of the metallic domains is significantly smaller than the size in the works presented above.

#### Other applications

The ability to controllably tailor the properties of SAMs, in combination with the specific properties of titanium dioxide, which include photocatalytic activity and superhydrophilicity, provides a platform for a wide range of applications. Among these, one may highlight the UV-protected polymeric materials based on amorphous TiO_2_ grown on sulfonated SAMs attached to polymeric sheets [115]. Another application is the use of hydrophobic SAMs (1*H*,1*H*,2*H*,2*H*-perflurooctyl-triethoxysilane) attached to TiO_2_ on titanium to improve the blood compatibility of titanium-based biomedical devices and implants [116]. A different application is the prevention of pitting corrosion by the highly uniform films of TiO_2_ grown on sulfonate-terminated SAMs [117].

## Conclusion

The ability to control the properties of self-assembled monolayers (SAMs) attached to solid surfaces and the unusual photocatalytic properties of titanium dioxide provide a rationale for studying systems comprising of both. Such systems can be realized in the form of SAMs grown on TiO_2_ or, in a complementary manner, as TiO_2_ grown on SAMs.

This mini-review summarizes the current knowledge on SAMs attached to titanium dioxide while focusing on the resemblances and differences between SAMs on titania and SAMs on the more frequently studied substrate of silica. Among the differences one finds the use of sulfonic acid headgroups and the faster chemisorption of alkylsilane monolayers.

Mastering micropatterning is a key issue en route to the successful assimilation of a variety of titanium dioxide based devices. Accordingly, particular attention was given to describing a variety of methods and techniques aimed at exploiting the photocatalytic properties of titanium dioxide for patterning. Reports on a variety of applications were discussed. The examples portrayed above, representing the areas of photovoltaics, microelectronics, microelectromechanics, photocatalysis, corrosion prevention and even biomedicine should be regarded as appetizers, paving the way for further studies to be performed.
